# Malignant Evaluation and Clinical Prognostic Values of M6A RNA Methylation Regulators in Prostate Cancer

**DOI:** 10.7150/jca.55140

**Published:** 2021-04-24

**Authors:** Qijie Zhang, Jiaochen Luan, Lebin Song, Xiyi Wei, Jiadong Xia, Ninghong Song

**Affiliations:** 1 Department of Urology, The First Affiliated Hospital of Nanjing Medical University, Nanjing, China.; 2 Department of Dermatology, The First Affiliated Hospital of Nanjing Medical University, Nanjing, China.; 3 The Affiliated Kezhou People's Hospital of Nanjing Medical University, Kezhou, Xinjiang, China.

**Keywords:** prostate cancer, m6A RNA methylation, prognosis, gene signature.

## Abstract

**Objective:** M6A RNA modification is closely associated with tumor genesis and progression of several malignancies; however, its role in prostate cancer (PCa) remains poorly understood.

**Materials and methods:** Expression data and corresponding clinicopathologic information were available freely from the Cancer Genome Atlas (TCGA) dataset. We compared the expression level of m6A RNA methylation regulators in PCa with different clinicopathologic characteristics and identified subgroups based on their expressions with consensus clustering. To build the signature and assess its prognostic value, several methods were used for the analysis, including univariate Cox regression analysis, Least Absolute Shrinkage and Selection Operator (LASSO) regression analysis, time-dependent receiver operating curve (ROC), and Kaplan-Meier (KM) survival analysis.

**Results:** Most of the m6A RNA methylation regulators were differentially expressed not only between normal and tumor tissue but also among PCa stratified by different clinicopathologic characteristics. There were obvious differences between two clusters, cluster 1 and 2, regarding clinicopathologic features, and the recurrence-free survival (RFS) in cluster 2 was significantly worse than cluster 1. We developed an eleven-gene signature which exhibited a high prognostic value and was able to independently predict RFS. Moreover, a nomogram which integrated clinical information and the gene signature was capable of distinguishing high-risk recurrent patients.

**Conclusion:** These methylation regulators are correlated to clinicopathologic characteristics in PCa and a prognostic model using m6A methylation-related genes is constructed and of high predictive value for recurrence after RP.

## Introduction

As one of the most prevalent malignant tumors, prostate cancer (PCa) is the second most common cause of cancer-related death among males in the Western countries 1-3. According to the American Cancer Society statistic 2020, it is estimated that approximately 191,930 newly PCa patients are diagnosed and there are 33,000 deaths in the USA in 2020, accounting for 21% for incidence and 10% for mortality of all tumor cases 3. Early localized PCa carries a 5-year survival rate of nearly 100%, while the rate drops to about 30% in advanced PCa 4. As is known, PCa patients are treated with surgery, chemotherapy, hormone therapy, or radiation, and currently, radical prostatectomy (RP) remains the primary curative treatment 5-7. Nevertheless, approximately 20% of patients following RP will suffer from recurrence within 10 years and may eventually develop to castration-resistant prostate cancer (CRPC) 8, 9. If patients progress into CRPC, the treatment will be limited and the survival time will decrease, though some novel drugs such as enzalutamide and abiraterone are clinically taken 10, 11. Hence, it is significant to identify PCa patients at high risk of recurrence following RP for the optimal management and surveillance. Nowadays, the prediction of recurrence is mainly based on several clinicopathologic factors, such as tumor node metastasis (TNM) stage, prostate specific antigen (PSA) level, Gleason score (GS) and surgical margin 12-14. However, on account of its heterogeneity, patients with the similar clinical parameters might develop into opposite results. Therefore, more effective, accurate and improved prognostic markers are urgently required to classify PCa patients into different risk categories.

In eukaryotic mRNA, N6-methyladenosine (m6A) is the most abundant and common posttranscriptional modification 15, 16. Accumulating evidence demonstrates that m6A RNA methylation can regulate a variety of biological processes, like mRNA splicing, stability, translation and intracellular distribution, and the dysregulation of m6A may result in cell death, decreased cell proliferation and developmental defects 17-19. The biological processes of m6A RNA modification are invertible and variable, which are under the control of methyltransferases, demethylases, and binding proteins 20. Methyltransferases (writers), including RBM15, ZC3H13, KIAA1429, WTAP, METTL3 and METTL14, can mediate the cellular m6A status, upregulate the m6A level, and form the multi-subunit methyltransferase enzyme complex^21^. Binding proteins (readers), containing HNRNPC, YTHDF2, YTHDF1, YTHDC2 and YTHDC1, can identify the modified site and transform the information of m6A RNA methylation into functional signals 22. ALKBH5 and FTO are categorized as demethylases (erasers), which can target RNA specifically 23. These findings can help to unravel the potential mechanism and the role of m6A RNA methylation in the regulation of gene expression at the post-transcriptional level. Increasing studies illustrate that m6A RNA modifications are tightly linked to the pathogenesis of several diseases, such as obesity, neuronal disorders, infertility and immunological diseases 24. Moreover, m6A RNA methylation participates in cell fate and cancer self-renewal 25. Although many researches have suggested that m6A RNA methylation were associated with proliferation, differentiation, invasion and metastasis in several types of malignancies, like pancreatic cancer, acute myeloid leukemia and hepatocellular carcinoma 26-28, the roles of these regulators in PCa regarding recurrence remain less understood and need to be completely explored.

In our study, we comprehensively analyzed the relationship between the expression of these regulators and clinicopathologic factors and their prognostic roles in PCa.

## Materials and Methods

### Data sources

In our study, 499 localized PCa patients following RP from two datasets were included. One was from the Cancer Genome Atlas (TCGA), and another was from the Gene Expression Omnibus (GEO). The RNA-seq transcriptome data and corresponding clinicopathologic information of 393 patients were downloaded from the TCGA dataset (https://www.ncbi.nlm.nih.gov/geo/). The RNA-seq data along with clinical information of 106 patients were available in GSE54460. Inclusion criteria of patients enrolled in this study were as follows: (I) the clinicopathological parameters, such as age, Gleason score (GS), clinical stage T (cT), pathological T (pT) stage or pathological N (pN) stage were included; (II) the outcomes (overall survival (OS) or biochemical recurrence (BCR)) of patients were contained; (III) the transcriptome profiling data (RNA-seq data) of prostate cancer samples were included; (Ⅳ) at least 50 samples in each dataset. The exclusion criteria were as follows: (I) patients receiving chemotherapy or radiotherapy before RP; (II) insufficient sample volumes. We divided the patients in TCGA dataset into the training set and internal validation set with the ratio of 7:3, and GSE54460 was taken as the external validation dataset, of which detailed clinicopathological information was displayed in **Table 1**.

### Bioinformatic analysis

We firstly selected expression data of thirteen m^6^A RNA methylation regulators in the TCGA database, then systematically compared the expression level of these m^6^A RNA methylation regulators in PCa with different clinicopathologic characteristics. To explore the role of m^6^A RNA methylation regulators in PCa, we clustered patients into different groups with “ConsensusClusterPlus” (50 iterations, 80% item resampling, and Pearson correlation, http://www.bioconductor.org/). Principal component analysis (PCA) with the R package for R3.6.3 was used to exhibit the gene expression patterns in different PCa groups. Survival curves were generated to analyze the differences on OS and RFS between different groups with Kaplan-Meier method using the log-rank test. Moreover, the differences in clinicopathologic traits between groups were compared.

Considering the prognostic differences in RFS between groups, the Limma package (http://www.bioconductor.org/packages/release/bioc/html/limma.html) in R was used to identify the differentially expressed (DE) genes with the cut-off value of |log2 fold change (FC)|>1 and false discovery rate (FDR) <0.01. In the training set, univariate Cox regression analysis was performed to access the prognostic value of these DE genes. Next, the genes closely related to survival were selected to develop a potential signature with the least absolute shrinkage and selection operator (LASSO) Cox regression algorithm. According to the signature, each patient from the training set and validation set got their own risk score. Taking the median in the training set as the cut-off value, patients in both sets were classified into high- and low-risk group. The Kaplan-Meier survival curve and time-dependent receiver operating characteristic (ROC) curve were drawn to evaluate the predictive value of the signature. We also explored the relationship between the signature and clinicopathologic characters.

The nomogram model, which integrated the signature and clinicopathologic features, was built to predict survival in PCa patients as a quantitative tool. The calibration plot and time-dependent ROC analysis were used to investigate the calibration and the discrimination of the model.

### Statistics

The t-test or one-way ANOVA was used for continuous variables, and the chi-square test or Fisher exact for categorical variables. The Cox proportional hazards regression model was used to access the prognostic value of each parameter. All statistics were performed in IBM SPSS Statistics 22.0 (SPSS lnc.) and R software 3.6.3 (R Foundation for Statistical Computing, Vienna, Austria). Two-tailed P value < 0.05 was considered as statistically significant for all statistical analyses.

## Results

### Expression level of m6A RNA methylation regulators in PCa patients

**Figure 1:**
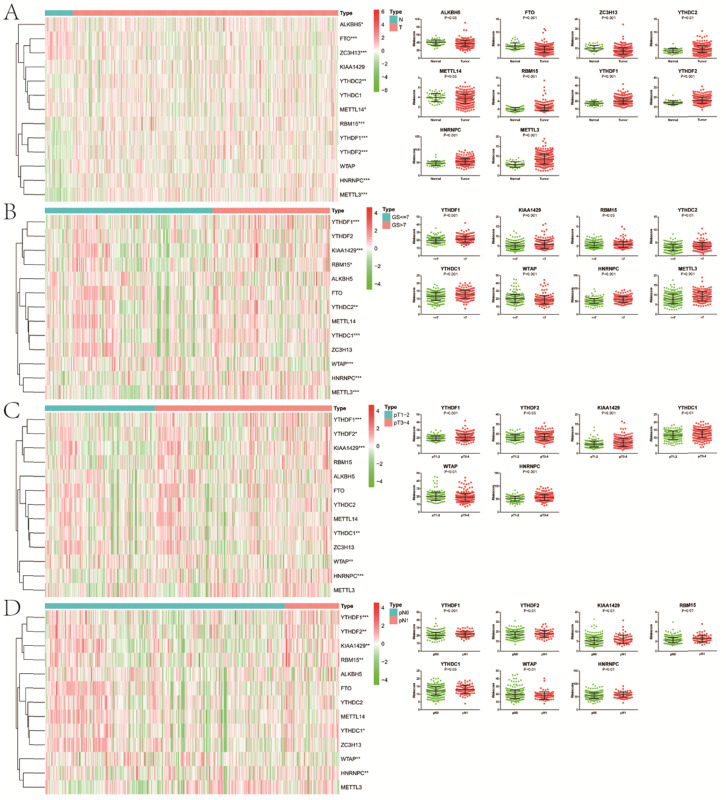
Expression of m6A RNA methylation regulators in PCa with different clinicopathologic characters. **A**, between normal and tumor tissue;** B**, between GS≤7 and GS>7; **C**, between pT1-2 and pT3-4; **D**, between pN0 and pN1. GS, Gleason score; pT, pathologic tumor; pN, pathologic lymph node.

Except for KIAA1429, YTHDC1 and WTAP, the remaining 10 m6A methylation regulators were differentially expressed between normal and tumor tissue (**Figure 1**). In the “readers” section, YTHDC2 (P<0.01), YTHDF1 (P<0.001), YTHDF2 (P<0.001) and HNRNPC (P<0.001) were all highly expressed in PCa. As for “writers”, ZC3H13 (P<0.001) and METTL14 (P<0.05) decreased significantly in PCa, while the opposed results appeared in RBM15 (P<0.001) and METTL3 (P<0.001). In terms of “erasers”, both ALKBH5 (P<0.05) and FTO (P<0.001) had a lower expression in PCa. Moreover, as GS increased, higher expression of YTHDF1 (P<0.001), KIAA1429 (P<0.001), RBM15 (P<0.05), YTHDC2 (P<0.01), YTHDC1 (P<0.001), HNRNPC (P<0.001) and METTL3 (P<0.001) appeared. Compared with pathological T1/2, there was an obvious increase in the expression of YTHDF1 (P<0.001), YTHDF2 (P<0.05), KIAA1429 (P<0.001), YTHDC1 (P<0.01), HNRNPC (P<0.001) in pathological T3/4. Similarly, the expression of FTHDF1 (P<0.001), YTHDF2 (P<0.01), KIAA1429 (P<0.01), RBM15 (P<0.01), YTHDC1 (P<0.05) and HNRNPC (P<0.01) in pathological N1 was significantly higher than that in pathological N0. However, we also noticed that lower WTAP was associated with higher GS (P<0.001), pT (P<0.01) and pN (P<0.01). Additionally, consistent results were observed in GSE54460 dataset for validation (**Figure S1A,B**).

### Interaction and correlation among m6A RNA methylation regulators

**Figure 2:**
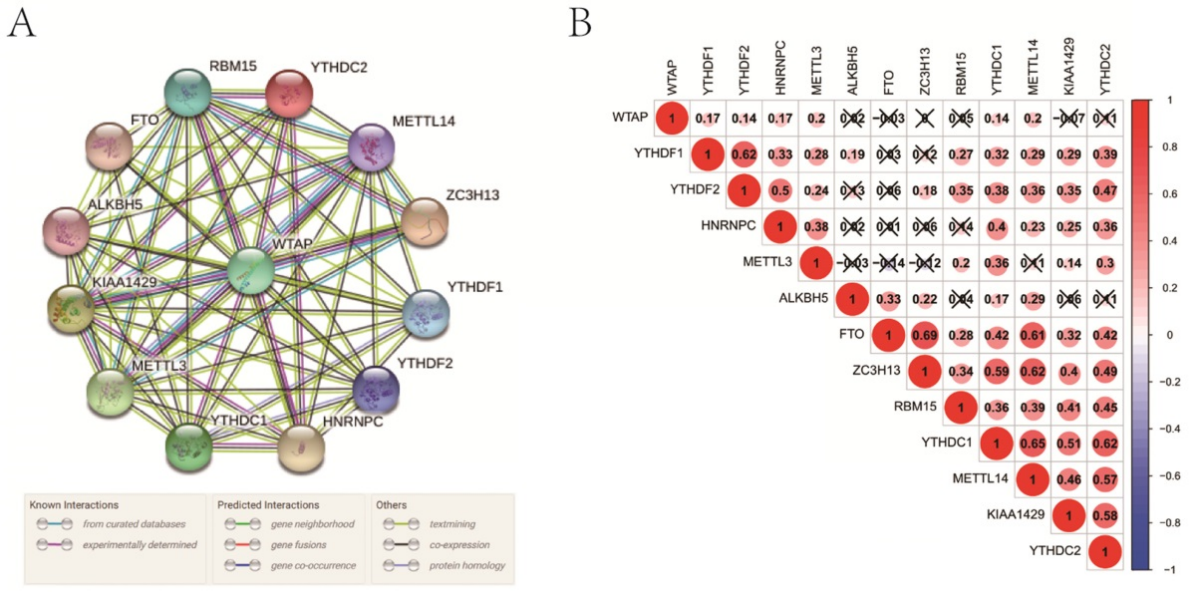
Interaction among m6A RNA methylation regulators. **A**, interaction network; **B**, correlation analysis.

In the interaction network, WTAP was at the center and interacted with other regulators mainly based on currently known and predicted interactions (**Figure 2A**). Especially, its interaction with some regulators, such as RBM15, YTHDC2, METTL14, ZC3H13, HNRNPC, YTHDC1, METTL3, and KIAA1429, had been supported by experimental evidence. In PCa, WTAP was positively correlated with some “writers” including METTL3 and METTL14 (**Figure 2B**). As for “readers”, YTHDF1, YTHDF2, HNRNPC, YTHDC1, and YTHDC2 were closely positively correlated with each other. In the “erasers”, a significant correlation was observed between FTO and ALKBH5 as well. In addition, there were obvious correlations among “writers”, “readers”, and “erasers”.

### Consensus clustering for category identification

**Figure 3:**
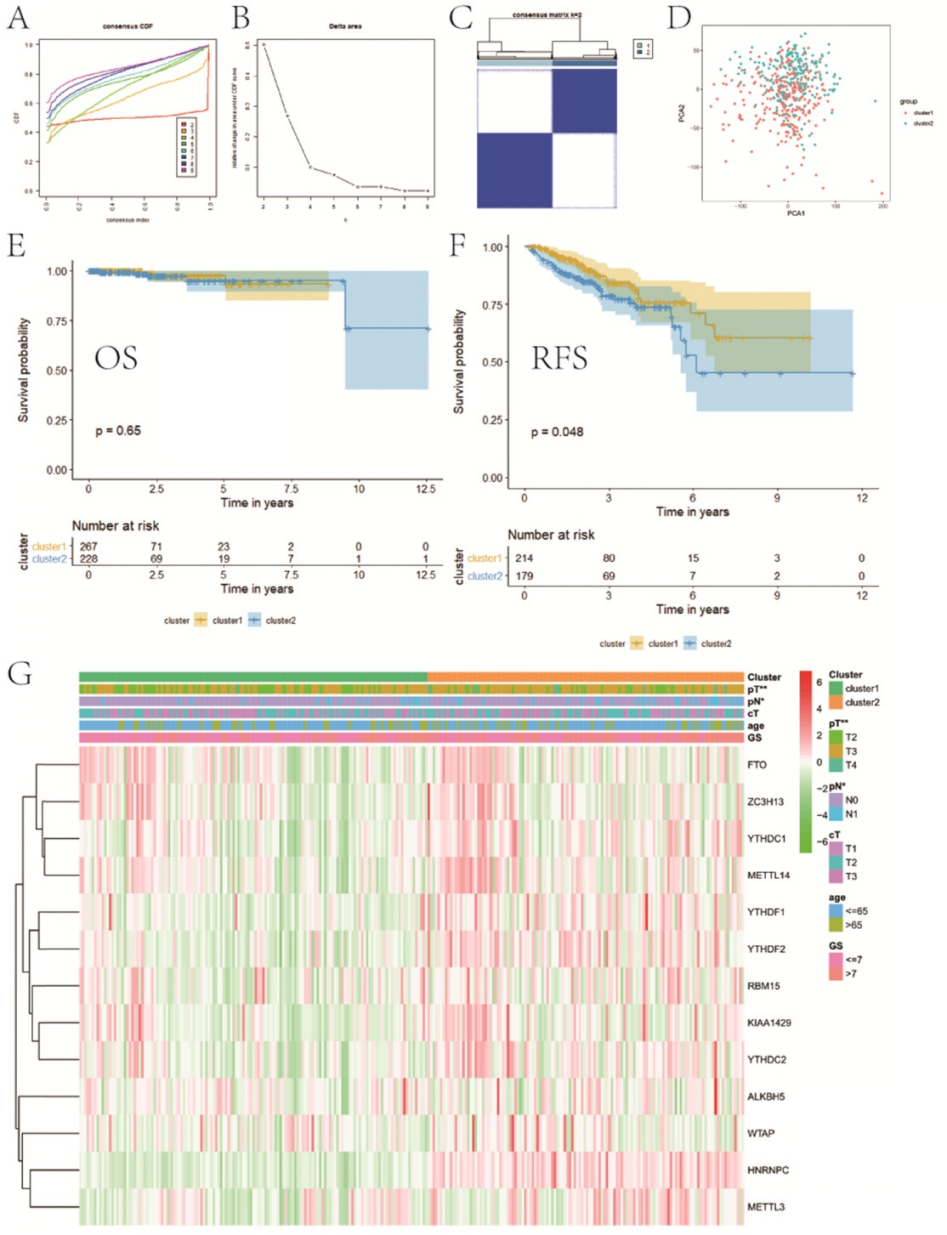
Consensus clustering for category identification. **A**, consensus clustering CDF for k=2 to 9; **B**, relative change in area under CDF curve for k=2 to 9; **C**, consensus clustering matrix for k=2; **D**, principal component analysis of the total RNA expression profile; **E**, KM curve of OS between two clusters; **F**, KM curve of RFS between two clusters; **G**, heatmaps and clinicopathologic characters of two clusters. CDF, cumulative distribution function; KM, Kaplan-Meier; OS, overall survival; RFS, recurrence-free survival. *P<0.05, **P<0.01.

**Figure 4:**
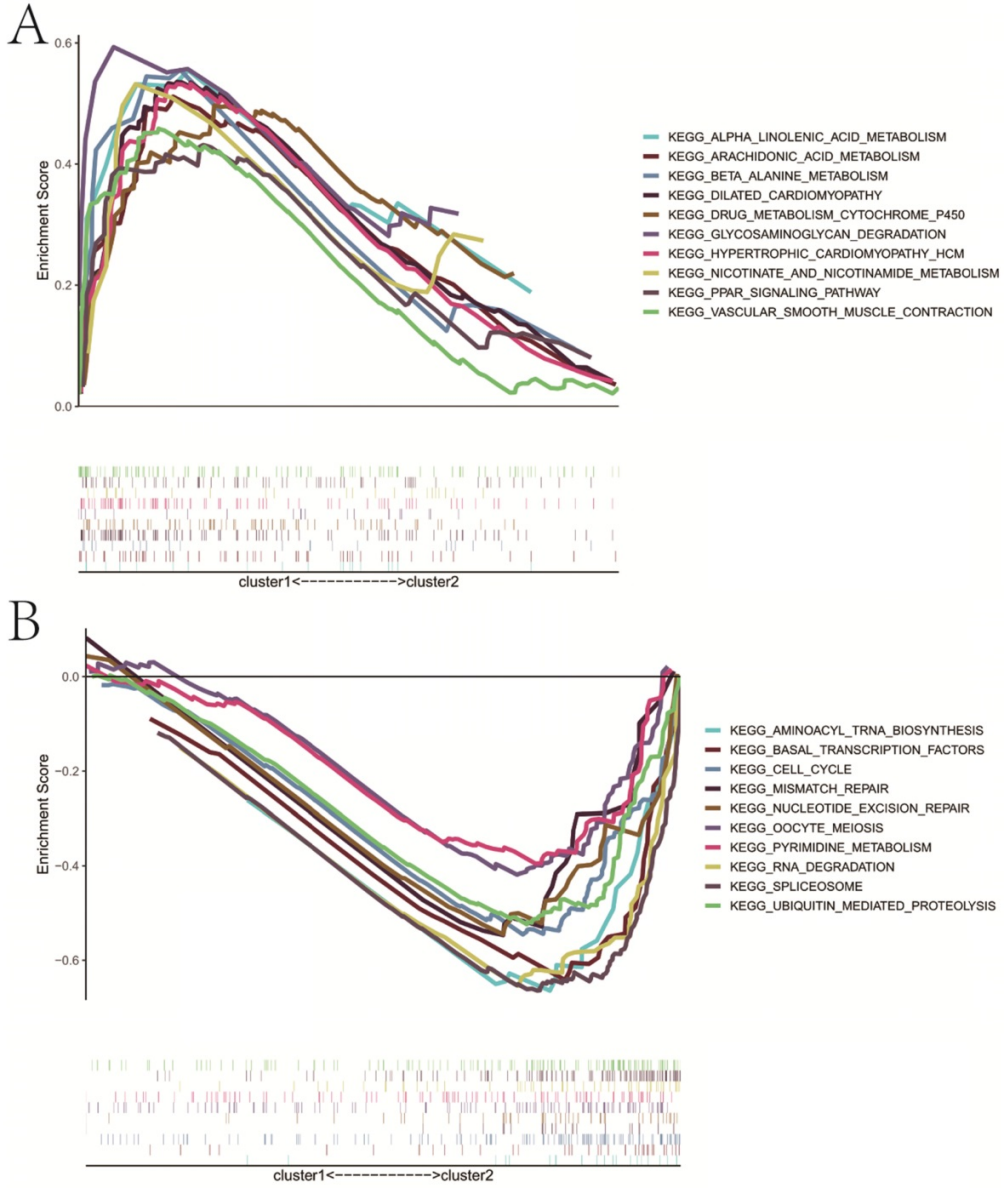
Stratified GSEA analysis. **A**, KEGG pathways enriched in cluster 1; **B**, KEGG pathways enriched in cluster 2. GSEA, gene set enrichment analysis.

Based on the expression similarity of m6A RNA methylation regulators, we chose k = 2 as a suitable value with clustering stability accumulating from k =2 to 9 (**Figure 3A-C**). The result of PCA showed that a relatively evident distinction existed between two subgroups, cluster 1 and cluster 2 (**Figure 3D**). Moreover, patients in cluster 1 had a better RFS (P<0.05) than those in cluster 2, while no differences on OS were observed (P>0.05) (**Figure 3E,F**). Compared with cluster 1, patients in cluster 2 had a significantly higher pT (P<0.01) and pN (P<0.05) (**Figure 3G**). However, there were no differences on clinical T stage, age and GS between two clusters. In addition, stratified GSEA revealed that some pathways, such as PPAR signaling pathway and arachidonic acid metabolism, were enriched in cluster 1, while some different pathways, like cell cycle, RNA degradation, spliceosome, and basal transcription factors, were associated with cluster 2 (**Figure 4A,B**).

### Construction of a prognostic signature for RFS based on DE genes between two clusters

**Figure 5:**
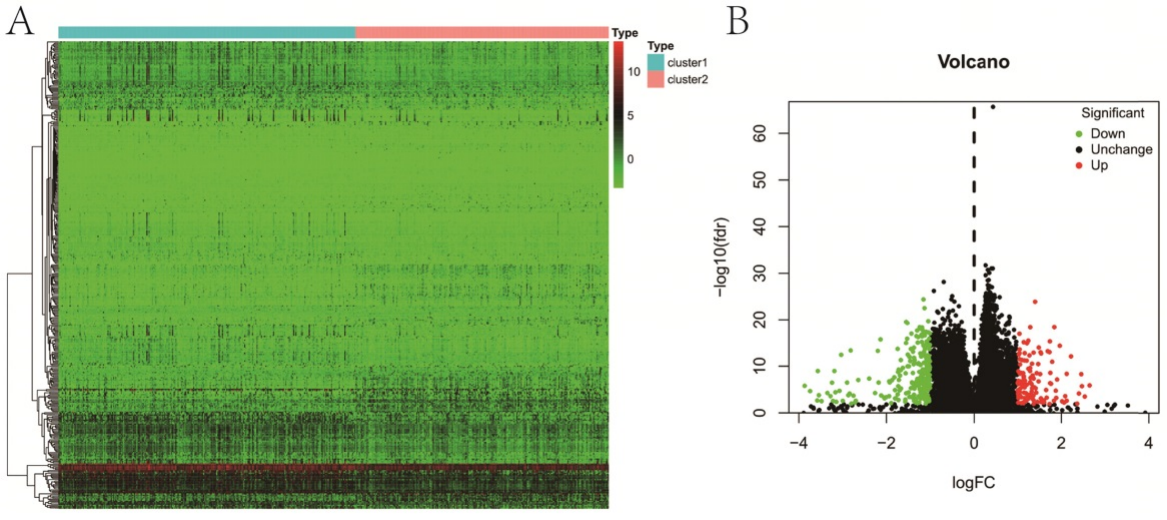
Differential expression analysis between two clusters. **A**, heatmap; **B**, volcano map.

**Figure 6:**
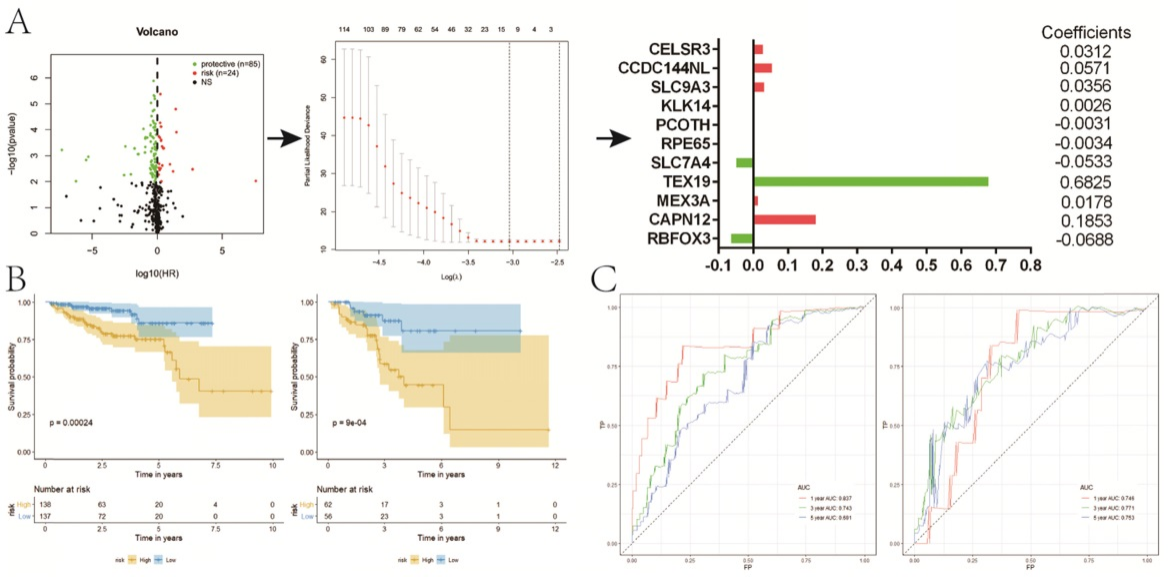
Construction of a prognostic signature for RFS based on DE genes between two clusters. **A**, the process of building the signature; **B**, KM curves of RFS for patients in the training (left) and validation (right) set; **C**, ROC curves for 1, 3, 5-year survival prediction by the risk signature. RFS, recurrence-free survival; DE, differentially expressed; KM, Kaplan-Meier.

A total of 363 DE genes were identified, of which 235 genes were up-regulated and the remaining 128 genes were down-regulated in cluster 1 (**Figure 5A,B**). We found that 109 of 363 DE genes were significantly associated with RFS, including 85 protective genes with HR<1 and 24 risk genes with HR>1 (**Figure 6A**). Then, 109 prognostic genes were used to develop a signature based on the LASSO Cox regression algorithm in the training set. Eventually, eleven genes were enrolled to construct the risk signature according to the minimum criteria. Based on the coefficients obtained from the LASSO algorithm, the following formula was used to calculate the risk score: [(0.0312)*CELSR3 expression] + [(0.0571)*CCDC144NL expression] + [(0.0356)*SLC9A3 expression] + [(0.0026)*KLK14 expression] - [(0.0031)*PCOTH expression] - [(0.0034)*RPE65 expression] - [(0.0533)*SLC7A4 expression] + [(0.6825)*TEX19 expression] + [(0.0178)*MEX3A expression] + [(0.1853)*CAPN12 expression] - [(0.0688)*RBFOX3 expression]. Patients in low-risk group had a significantly better RFS than those in high-risk group in both sets (P<0.001, P<0.001, respectively) (**Figure 6B**), which was validated in GSE54460 dataset (P=0.011) (**Figure S1C**). Moreover, the powerful predictive value of the risk score was noticed in both sets (**Figure 6C**).

### Association of gene signature with clinicopathological characters and m6A regulators in PCa

**Figure 7:**
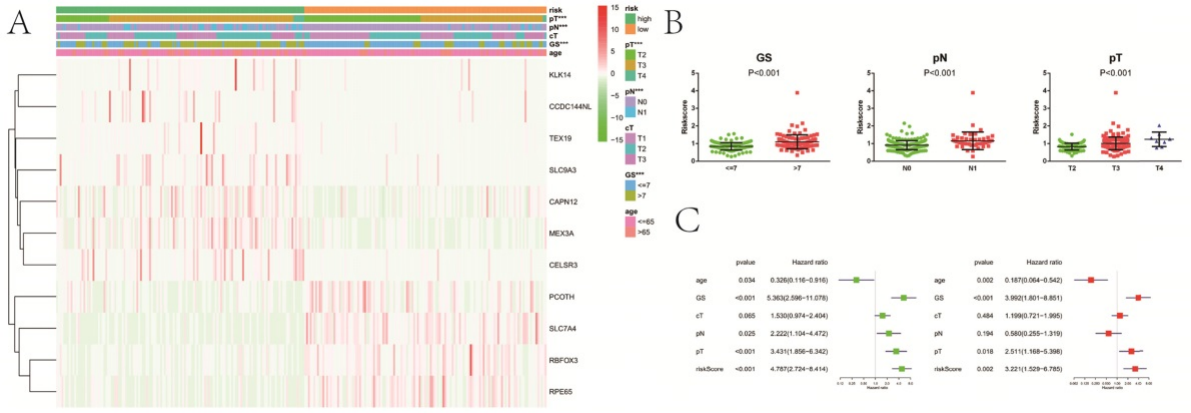
Relationship between the risk signature and clinicopathologic characters. **A**, heatmap showing clinicopathologic characters between low- and high-risk group; **B**, distribution of risk scores in PCa patients stratified by GS, pT and pN; **C**, univariate and multivariate Cox regression analysis for RFS in PCa patients. GS, Gleason score; pT, pathologic tumor; pN, pathologic lymph node; cT, clinical tumor; RFS, recurrence-free survival. ***P<0.001.

**Figure 8:**
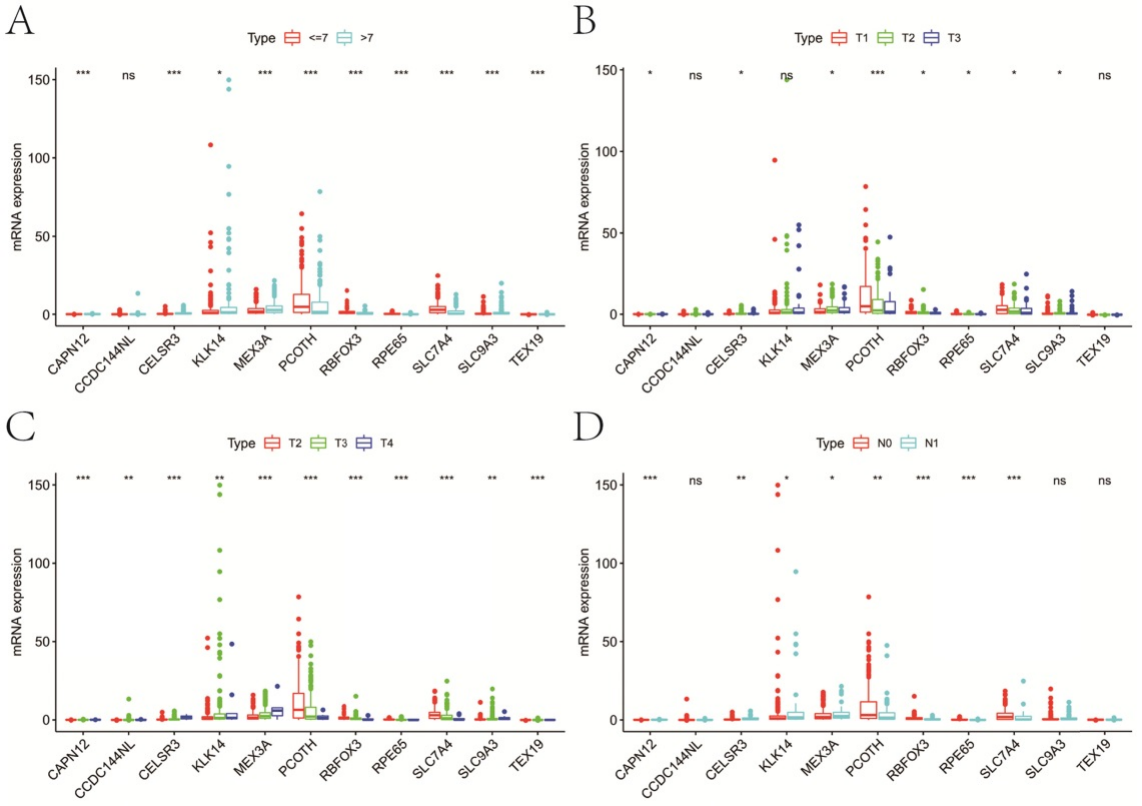
Expression of genes in the signature at different clinicopathologic characters. **A**, GS; **B**, cT; **C**, pT; **D**, pN. GS, Gleason score; pT, pathologic tumor; pN, pathologic lymph node; cT, clinical tumor; ns, not significant. *P<0.05, **P<0.01, ***P<0.001.

Compared with patients in low-risk group, high-risk patients had a significantly higher GS (P<0.001), pT (P<0.001) and pN (P<0.001) (**Figure 7A**). There were no obvious differences on clinical T stage and age between low- and high-risk groups. We noticed that risk score significantly rose when GS (P<0.001), pT (P<0.001) and pN (P<0.001) increased (**Figure 7B**). Similarly, risk score increased with the elevation of GS (P=0.001) and psa (P=0.001) (**Figure S1D,E**). In the univariate Cox analysis, age (P<0.05), GS (P<0.001), pN (P<0.05), pT (P<0.001), and riskScore (P<0.001) were closely associated with RFS (**Figure 7C**). Multivariate Cox analysis presented that age (P<0.05), GS (P<0.001), pT (P<0.05), and riskScore (P<0.01) remained significantly linked with RFS, which indicating that gene signature could serve as an independent prognostic element for RFS in PCa. It also demonstrated that most of genes in the signature were differentially expressed in groups with different GS, cT, pT and pN (**Figure 8**). Moreover, part of the genes in the signature, except CCDC144NL and TEX19, were significantly correlated with some m6A regulators (**Table 2**).

### Combination with clinicopathological variables to build a predictive nomogram

**Figure 9:**
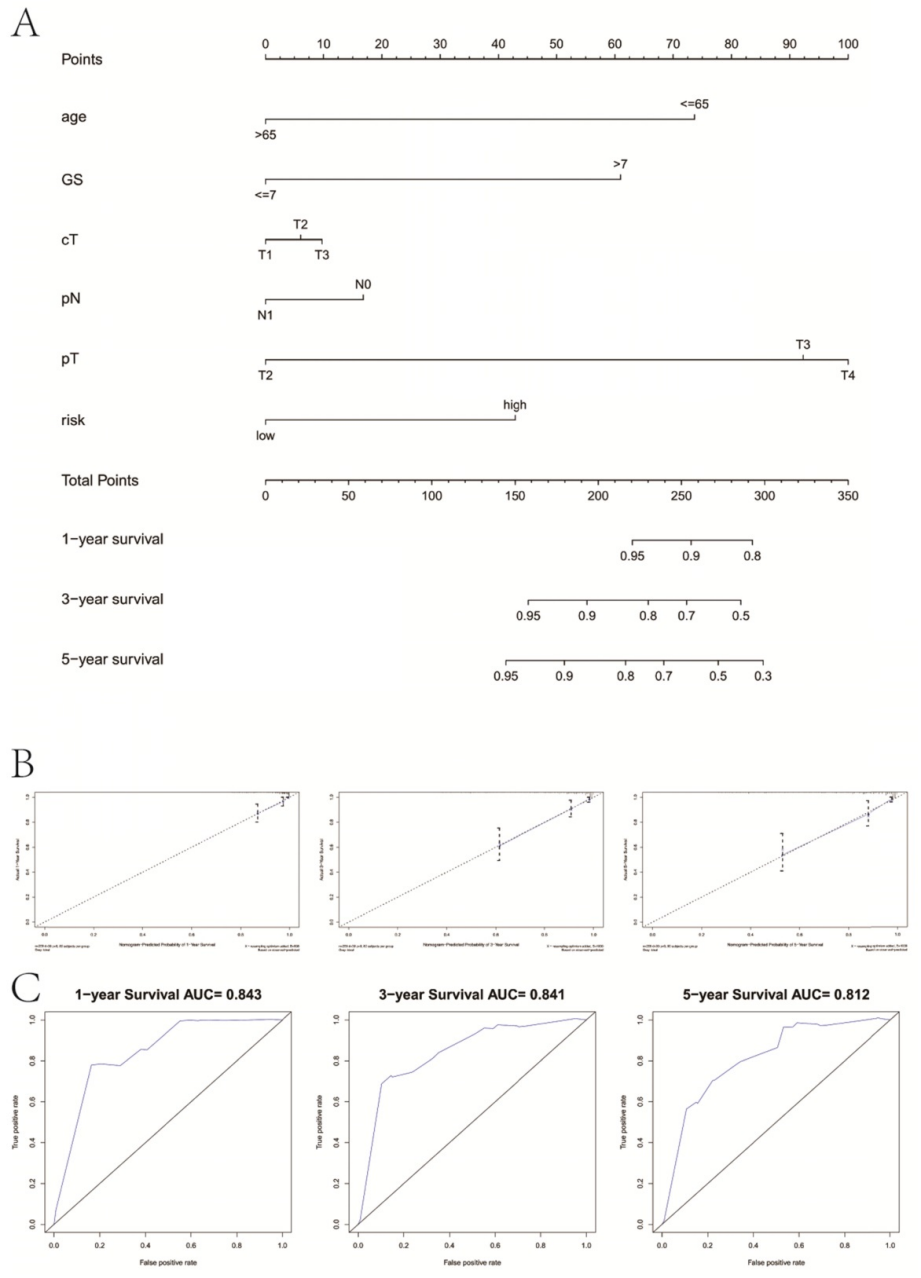
Combination with clinical variables to build a predictive nomogram. **A**, Nomogram plot to predict 1-, 3- and 5-year survival; **B**, Calibration plot of the nomogram to predict 1-, 3- and 5-year survival; **C**, ROC curves of the nomogram to predict 1-, 3- and 5-year survival. GS, Gleason score; pT, pathologic tumor; pN, pathologic lymph node; cT, clinical tumor.

We drew a nomogram plot to quantify the possible risk of RFS in PCa by integrating the gene signature with clinicopathological information (age, GS, cT, pN, pT) (**Figure 9A**). This allowed us to calculate the estimated possibility of recurrence in PCa patients at 1, 3 and 5 years by plotting a vertical line between the total points and each prognosis axis. The AUC value of 1-year, 3-year and 5-year recurrence of nomogram was 0.843, 0.841 and 0.812, respectively (**Figure 9B**). Calibration curves of the nomogram showed no deviations from the reference line and no recalibration required (**Figure 9C**).

## Discussion

PCa is one of the most prevalent tumors among elderly males 3. More than half of patients will choose RP as the primary treatment 29-31. After the surgery, a significant proportion of PCa patients may experience recurrence, including biochemical recurrence, locoregional recurrence, distant metastasis, and new primary tumor. However, some patients with indolent PCa should be treated without immediate therapies, which may have few effects on the living quality. Therefore, to propose the early intervention to recurrent PCa and to avoid unnecessary overtreatment of indolent PCa, it is important to identify a more precise predictive risk stratification tool to classify patients into different risk categories after RP.

Studies have demonstrated that traditional epigenetics have different kinds of functions in PCa carcinogenesis, metastasis and outcomes 32, 33. In our study, we systematically analyzed another area of epigenetics, namely m6A RNA methylation, and their prognostic value for recurrence in localized PCa patients for the first time. Firstly, we compared the expression value of m6A RNA methylation regulators in PCa and control tissues. Then, we comprehensively explored the association between these regulators and clinicopathological variables in PCa. Among these regulators, Cai, et al. pointed out that METTL3, the m6A methyltransferase, was overexpressed in PCa cell lines, and via the hedgehog pathway, METTL3 contributed to the growth and motility of PCa cells 34. Similarly, in our study, we found that compared to normal tissue, METTL3 expressed higher in PCa. In addition, as GS increased, higher expression of METTL3 would appear, which indicated that METTL3, serving as one of the 'writers', might made a great contribution to the growth and progression of PCa. WTAP, another 'writer', was the center and interacted with other regulators. Recently, WTAP displays oncogenic activities in different tumors, such as acute myeloid leukemia and serous ovarian cancer 35, 36. Melissa, et al. illustrates that WTAP upregulation has an oncogenic effect only when a functional METTL3 exists 37. The regulation of WTAP might be controlled by METTL3 in direct and indirect manners, such as mRNA translation and stability. Meanwhile, in our analysis, WTAP was positively connected with METTL3 according to the interaction network. The two 'erasers', FTO and ALKBH5, had a low expression in PCa. Liu, et al. claims that FTO affects cellular energy metabolism in breast cancer, such as lactic acid, adenosine triphosphate, and hexokinase activity via the PI3K/AKT signaling 38. As reported by Pu, et al, ALKBH5 expression is obviously elevated in endometrial cancer, and through erasing IGF1R mA-modifications, ALKBH5 positively regulates proliferation and invasion of cancer 39. With respect to 'readers', both YTHDF1 and YTHDC1 were linked to higher GS, pathological T and pathological N. YTHDF1 is overexpressed in the colorectal cancer (CRC), and inhibition of Wnt/β-catenin pathway can be achieved by YTHDF1 silencing in CRC cells^40^. A case-control study from 7 centers about Chinese children shows that polymorphisms of the YTHDC1 gene have been associated with increased risk of hepatoblastoma^41^. Taken together, similar to other tumors, these regulators may participate in pathological processes in PCa, which uncovers the mechanism of regulators in the development of tumors from a different perspective.

By utilizing the consensus clustering, localized PCa patients were divided into two subgroups (cluster 1 and 2) based on the expression of 13 regulators. Marked differences in the RFS and several clinicopathological parameters, such as pT and pN, were identified between two subgroups, which suggested that these regulators expression were dramatically linked to the features of PCa. Additionally, stratified GSEA also illustrated that two subgroups were enriched in different pathways. Further, a total of 363 DE genes were identified between two clusters, and 11 eligible DE genes (CELSR3, CCDC144NL, SLC9A3, KLK14, PCOTH, RPE65, SLC7A4, TEX19, MEX3A, CAPN12 and RBFOX3) were eventually selected to establish the gene signature that could stratify PCa patients into different risk categories. The patients in the low-risk category had a significantly better RFS than those at high risk, in both training and validation set. By Cox regression analysis the risk score emerged as an independent prognostic element for recurrence in localized PCa patients. Next, it showed that patients at high risk exhibited higher GS, pT, and pN, and with the increase of GS, pT and pN, the risk score had a tendency to rise. Besides, we also found that the candidate DE genes, except CCDC144NL and TEX19, had a strong association with several m6A RNA modification regulators. Among these candidate DE genes, some studies indicated that they played primary roles in tumorigenesis and development, even in PCa. For example, PCOTH is overexpressed in PCa cells, and through the TAF-Ibeta pathway, it takes part in the growth and survival of PCa cells, which might be a potential therapeutic target for PCa treatment 42. Regarding KLK14, it is reported that KLK14 demonstrates key modulatory roles in advanced PCa 43. Highly expressed KLK14 is an indicator for poor prognosis, and its gene polymorphisms are remarkably linked to PCa aggressiveness 44. TEX19, known as one of the cancer/testis (CT) genes, might drive cell proliferation in a variety of cancers through the oncogenic transcript regulation mechanism. Planells-Palop et al. displays that TEX19 expression might become a new tumor biomarker and have the broad-spectrum potential to provide the cancer-specific therapeutic target 45. Luo discovers that RBFOX3 promotes cell division and invasion, and improves migratory ability in gastric cancer 46. In addition to the above-mentioned genes, other genes play some roles in the progression and prognosis of tumors, such as CELSR3 in hepatocellular carcinoma 47, CCDC144NL in gastric cancer 48, RPE65 in nonmelanocytic skin tumor 49, SLC27A4 in lung cancer 50, MEX3A in bladder urothelial carcinoma 51, CAPN12 in laryngeal cancer 52 and SLC4A4 in breast cancer 53. In a word, the functional roles and underlying mechanisms of 11 eligible DE genes in the signature, and their association with m6A methylation regulators in PCa are still need further researches.

It is worth noting that our study suffers from some limitations. One limitation is the retrospective design and relatively small sample size. Another limitation is lack of experimental verification. Despite aforementioned limitations, the role of m6A RNA regulators and the prognostic value of our signature for RFS in PCa cannot be denied.

**Figure 10:**
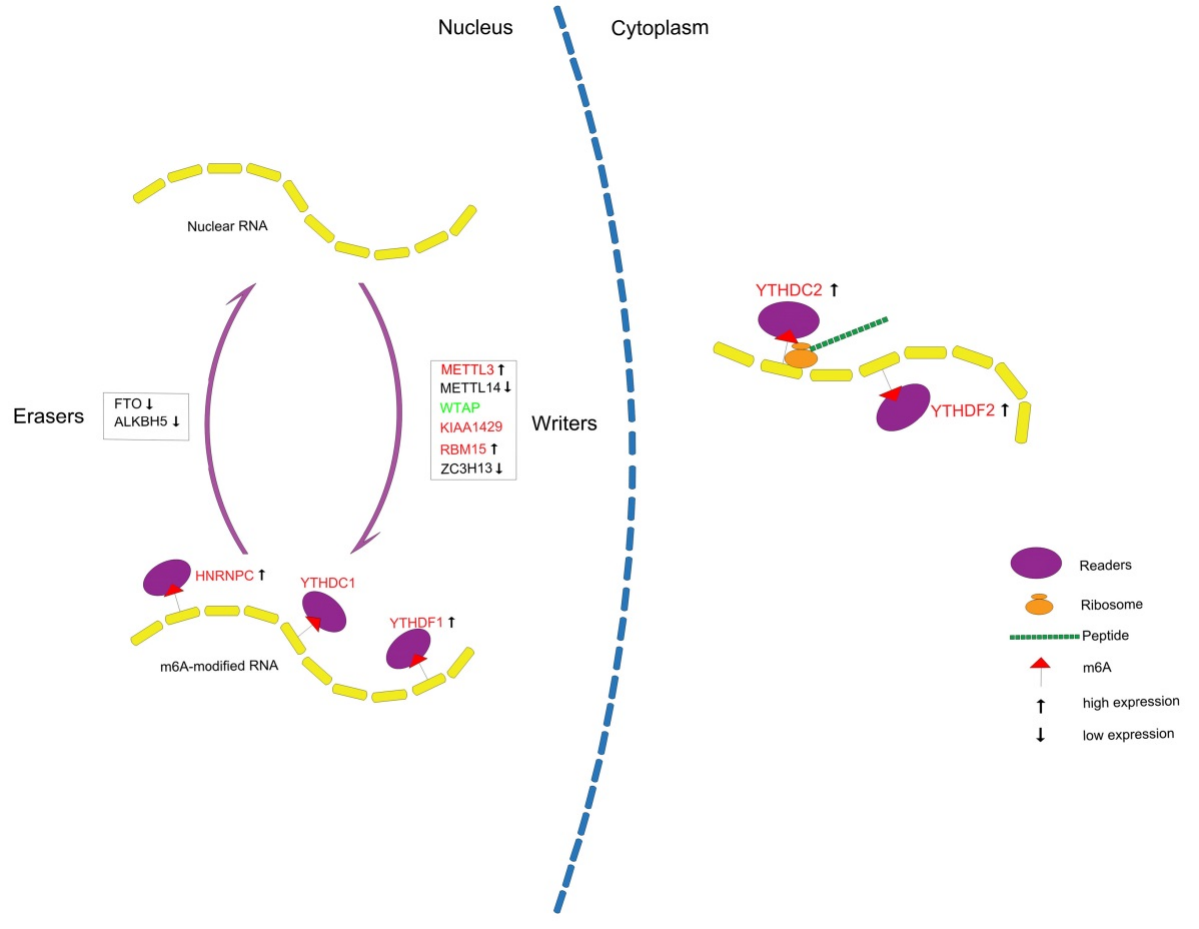
Summary for the expression levels and potential roles of m6A RNA methylation regulators in PCa. The red and green represent the risk and protective gene, respectively.

In conclusion, we systematically analyzed the expression of m6A RNA methylation regulators in PCa (**Figure 10**) and constructed a prognostic model with high predictive value based on m6A methylation-related genes for recurrence after RP, which might contribute to a better understanding of the role of m6A RNA methylation in PCa.

## Supplementary Material

Supplementary figure S1.

## Figures and Tables

**Table 1:** Clinicopathological features of patients involved in the training set and validation set.

		TCGA dataset		GSE54460
		training set	internal validation set	external validation set
Total cases		275	118	106
Age		61(41-78)	61(44-77)	na
GS	≤7	163	74	91
	>7	112	44	15
psa	<10	na	na	72
	≥10	na	na	31
	unknown	na	na	3
cT	T1	90	42	na
	T2	103	39	na
	T3	32	15	na
	unknown	50	22	na
pT	T2	103	49	na
	T3	160	68	na
	T4	8	1	na
	unknown	4	0	na
pN	N0	193	83	na
	N1	46	18	na
	unknown	36	17	na

GS, Gleason Score; cT, clinical T stage; pT, pathological T stage; pN, pathological N stage; na, not available.

**Table 2:** Correlation of the genes in the signature with m6A methylation regulators in PCa.

	KIK14	CCDC144NL	TEX19	SLC9A3	CAPN12	MEX3A	CELSR3	PCOTH	SLC7A4	RBFOX3	RPE65
FTO	-0.09	0.02	-0.14	0.09	-0.02	0.07	0.02	**-0.2**	-0.04	0.03	**0.2**
ALKBH5	-0.05	0.09	0.01	0.01	0.1	0.01	-0.03	-0.08	-0.05	0.11	0.15
METTL3	0.08	0.11	0.02	0.14	**0.22**	0.14	0.15	0.09	-0.11	**-0.35**	**-0.21**
METTL14	-0.05	0.09	-0.1	0.11	-0.01	0.09	-0.03	-0.15	0.01	-0.15	0.11
WTAP	-0.11	-0.04	-0.06	-0.09	-0.11	-0.1	-0.15	**0.17**	**0.24**	-0.1	0.05
ZC3H13	0.01	-0.04	-0.07	0.08	0.01	0.16	0.01	**-0.17**	0.01	-0.04	**0.19**
KIAA1429	**0.26**	0.03	-0.01	**0.22**	0.08	**0.39**	0.12	**-0.2**	**-0.19**	**-0.24**	-0.09
RBM15	0.02	0.1	0.05	0.08	0.08	**0.22**	0.13	-0.09	**-0.17**	**-0.29**	**-0.19**
YTHDF1	-0.02	0.1	0.09	0.1	**0.2**	**0.19**	**0.21**	**-0.2**	-0.11	**-0.36**	**-0.17**
YTHDF2	0.15	0.1	0.03	0.14	0.16	**0.45**	**0.19**	**-0.2**	**-0.19**	**-0.37**	**-0.18**
YTHDC1	0.07	0.02	-0.04	**0.2**	**0.23**	**0.28**	0.09	**-0.22**	-0.14	**-0.22**	0.01
YTHDC2	0.03	0.09	0.01	**0.2**	0.08	**0.25**	**0.18**	**-0.17**	**-0.2**	**-0.32**	-0.06
HNRNPC	**0.24**	0.11	0.11	**0.22**	**0.26**	**0.53**	**0.32**	**-0.27**	**-0.22**	**-0.38**	**-0.26**

Bold numbers mean P <0.05.

## Abbreviations

PCa: prostate cancer
TCGA: the Cancer Genome Atlas
LASSO: Least Absolute Shrinkage and Selection Operator
ROC: receiver operating curve
KM: Kaplan-Meier
RFS: recurrence-free survival
RP: radical prostatectomy
CRPC: castration-resistant prostate cancer
TNM: tumor node metastasis
PSA: prostate specific antigen
GS: Gleason score
cT: clinical stage T
pT: pathological T
pN: pathological N
OS: overall survival
BCR: biochemical recurrence
PCA: Principal component analysis
DE: differentially expressed
FC: fold change
FDR: false discovery rate
CRC: colorectal cancer
