# Supplementary material for: Malignant Evaluation and Clinical Prognostic Values of M6A RNA Methylation Regulators in Prostate Cancer
Source: J Cancer. 2021 Apr 24;12(12):3575–86. doi: 10.7150/jca.55140 (PMC8120168; doi:10.7150/jca.55140)
Supplement: Supplementary file 1 — Supplementary figure S1. [file jcav12p3575s1.pdf]

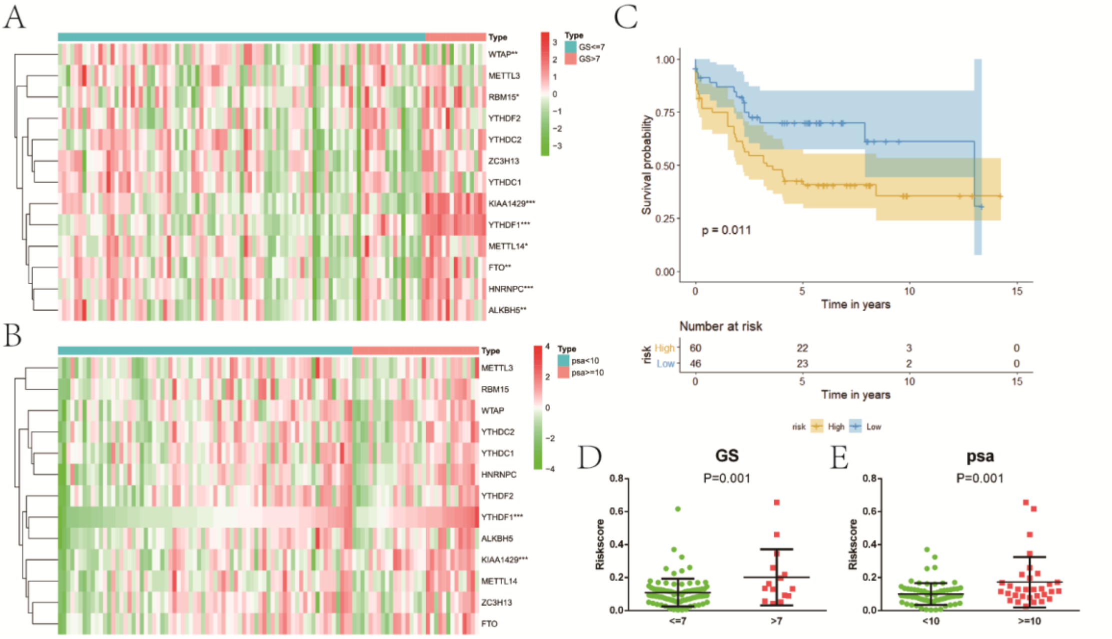

FigureS1. External validation results in GSE54460 dataset. **A-B**, differences in expression of m6A RNA methylation regulators regarding GS (A) and psa (B); **C**, KM curve of RFS between high-risk and low-risk group; **D-E**, distribution of risk scores in patients stratified by GS (D) and psa (E). GS, Gleason score; psa, prostate specific antigen; KM, Kaplan-Meier; RFS, recurrence-free survival.
